# Combination Effects of Clindamycin and Benzoyl Peroxide Against *Cutibacterium acnes*

**DOI:** 10.1111/1346-8138.70170

**Published:** 2026-02-09

**Authors:** Koyo Yoshihara, Shoji Seyama, Nobukazu Hayashi, Yuki Horiuchi, Waka Ishida, Takae Yasuda, Ikue Hou, Tokihiko Shimada, Saori Murakami, Mie Kakuta, Junichi Sugai, Masako Watanabe, Akiko Ishii, Mayumi Nomoto, Hiroko Ichimiya, Noriko Tanaka, Chikage Takeo, Kazuha Kasugai, Miwa Kobayashi, Utako Kimura, Masumi Kohno, Emi Nakazaki, Chiaki Murase, Yoshitsune Ban, Yuko Fukano, Yoshiki Miyachi, Hidemasa Nakaminami

**Affiliations:** ^1^ Department of Clinical Microbiology School of Pharmacy, Tokyo University of Pharmacy and Life Sciences Tokyo Japan; ^2^ Department of Dermatology Toranomon Hospital Tokyo Japan; ^3^ Akihabara Skin Clinic Tokyo Japan; ^4^ Waka Skin Care Clinic Niigata Japan; ^5^ Nakano Dermatology Hiroshima Japan; ^6^ Hou Dermatology Clinic Okayama Japan; ^7^ Shimada Clinic of Dermatology Kagoshima Japan; ^8^ Murakami Dermatology Clinic Ehime Japan; ^9^ Kakuta Clinic Tokyo Japan; ^10^ Sugai Dermatology Park Side Clinic Tochigi Japan; ^11^ Kobayashi Dermatology Clinic Hyogo Japan; ^12^ Tsukuba Suzuran Dermatology Clinic Ibaraki Japan; ^13^ NomotoMayumi Skincare Clinic Niigata Japan; ^14^ Ichimiya Dermatology Clinic Oita Japan; ^15^ Matsumoto Clinic Fukuoka Japan; ^16^ Yoyogiuehara Dermatology Clinic Tokyo Japan; ^17^ Kasugai Skin Clinic Aichi Japan; ^18^ Kobayashi Dermatology Clinic Fukuoka Japan; ^19^ Department of Dermatology Juntendo University School of Medicine Chiba Japan; ^20^ Sugimoto Dermatology Kanagawa Japan; ^21^ Emi Skin Clinic Shoto Tokyo Japan; ^22^ Nagoya Garden Clinic Aichi Japan; ^23^ Kirari Dermatology Clinic Gifu Japan; ^24^ Meguro Dermatology Clinic Tokyo Japan; ^25^ Graduate School of Public Health Shizuoka Graduate University of Public Health Shizuoka Japan

**Keywords:** antibiotic resistance, benzoyl peroxide, clindamycin, *Cutibacterium acnes*

## Abstract

This study aimed to evaluate the clinical efficacy of combination therapy with clindamycin and benzoyl peroxide in treating acne vulgaris. We assessed the antimicrobial susceptibility of *Cutibacterium acnes* isolates obtained from these patients. In addition, the potential risk of *C. acnes* developing resistance to clindamycin and benzoyl peroxide following exposure was investigated in vitro. We analyzed 182 *C. acnes* isolates from patients with acne to evaluate the clindamycin susceptibility and resistance determinants and to examine the association between topical clindamycin use and resistance. We also tested the resistance frequency of *C. acnes* to clindamycin monotherapy and clindamycin/benzoyl peroxide combination therapy in vitro. The clindamycin resistance rates in the clindamycin/benzoyl peroxide and clindamycin monotherapy groups were 22.9% and 46.7%, respectively. The combination group showed a significantly lower clindamycin resistance rate (*p* < 0.05). Under clindamycin monotherapy, resistant strains emerged at a frequency of 8.1 × 10^−8^ to 8.7 × 10^−8^, whereas no resistant strains were detected under clindamycin/benzoyl peroxide combination conditions. The combination of clindamycin and benzoyl peroxide effectively suppressed the emergence of clindamycin‐resistant *C. acnes*.

## 1 Introduction

*Cutibacterium acnes* is particularly abundant in the pilosebaceous area of the skin. The abnormal proliferation of occluded follicles induces inflammation and exacerbates acne vulgaris [1, 2]. Treatment depends on the severity of inflammatory lesions and the presence of comedones and involves the use of oral and/or topical antibiotics. Benzoyl peroxide has keratolytic and bactericidal properties. It generates free radicals during decomposition, reducing the emergence of antimicrobial‐resistant bacteria [3]. Topical antibiotics, such as clindamycin, are commonly used to treat mild acne. However, the prevalence of clindamycin‐resistant *C. acnes* among patients with acne has remarkably increased in recent years and has become a significant clinical problem [4, 5].

Macrolide and lincosamide resistance in *C. acnes* has been attributed to mutations in the 23S rRNA gene, as well as the acquisition of exogenous ribosomal RNA methyltransferase genes, such as *erm*(X) and *erm*(50) [6, 7, 8, 9, 10]. In contrast, benzoyl peroxide poses a low risk of selecting resistant strains and, to the best of our knowledge, this has not been reported. Combination formulations of clindamycin and benzoyl peroxide have been reported to exhibit synergistic bactericidal effects against *C. acnes* and high efficacy against resistant strains in Western countries [11]. Combination therapy may reduce the risk of the emergence of resistant strains during prolonged monotherapy. However, data on the current use of topical agents for the treatment of acne vulgaris and their association with clindamycin‐resistant *C. acnes* in Japan remain limited.

This study aimed to clarify the current use of topical agents for the treatment of acne vulgaris, and their relationship with antimicrobial resistance in *C. acnes*. Additionally, this study aimed to evaluate the effect of clindamycin monotherapy and clindamycin/benzoyl peroxide therapy on the emergence of clindamycin‐resistant strains in vitro.

## 2 Materials & Methods

### 2.1 Patients, Sample Collection, and Questionnaire Survey

From 2014 to 2023, a questionnaire regarding treatment history was administered to 182 patients who sought treatment for acne vulgaris at 26 medical facilities in Japan. Of these, 117 received benzoyl peroxide monotherapy, 30 received clindamycin monotherapy, and 35 received clindamycin/benzoyl peroxide combination therapy. A total of 182 *C*. *acnes* isolates obtained from patients who had used one of the three agents were included in the analysis.

### 2.2 Antimicrobial Susceptibility Testing and Detection of Resistance Determinants

Antimicrobial susceptibility testing was performed according to the Clinical and Laboratory Standards Institute (CLSI) guidelines for anaerobes using the twofold agar dilution method. The minimum inhibitory concentration (MIC) was determined as an indicator of susceptibility [12, 13]. Clindamycin hydrochloride (Tokyo Chemical Industry, Tokyo, Japan) was used as the test antibiotic. CLSI breakpoints have previously been used to interpret resistance. We detected the macrolide resistance genes [*erm*(X) and *erm*(50)] and analyzed mutations in the 23S rRNA gene using previously described polymerase chain reaction and sequencing methods [9, 10].

### 2.3 Frequency of Emergence of Resistant Strains

We determined the frequency of clindamycin‐resistant mutants using *C. acnes* reference strains ATCC 6919 and ATCC 11828. Modified GAM agar (AcuDia, Shimadzu, Japan) containing either clindamycin (8 μg/mL) or clindamycin (8 μg/mL) in combination with benzoyl peroxide (256 μg/mL) was used. The clindamycin concentration was determined according to the CLSI breakpoint, and the benzoyl peroxide concentration was set at its solubility limit in GAM agar. Each strain was cultured anaerobically at 37°C for 48 h, concentrated tenfold, spread onto plates, and incubated for 48 h to determine the number of colony forming units (CFU)/mL and to calculate the frequency of resistant mutants.

### 2.4 Ethical Approval and Patient Consent

This study was approved by the Ethics Committee of Tokyo University of Pharmacy and Life Sciences (approval no. HN‐D‐2023‐016). Written informed consent was obtained from all patients before sample collection.

### 2.5 Statistical Analysis

Statistical analyses were performed using the chi‐square (*χ* ^2^) test and the Students *t*‐test. A *p*‐value < 0.05 was considered statistically significant.

## 3 Results

### 3.1 Association Between Drug Use History, Clindamycin Resistance Rate, and Resistance Determinants

The clindamycin resistance rates differed significantly among the three treatment groups (*p* < 0.05) (Tables 1 and S1). The clindamycin monotherapy group (*n* = 30) showed the highest resistance rate (46.7%, *n* = 14). 23S rRNA mutations were detected in 20.0% (6/30 of the cases), and *erm*(X) and *erm*(50) were detected in 13.3% (4/30 of the cases), respectively. The resistance rate was 22.9% (*n* = 35) in the clindamycin/benzoyl peroxide group (*n* = 8). *erm*(50) was detected in 17.1% (*n* = 6) of patients and 23S rRNA mutations were detected in 5.7% (*n* = 2) of patients. *erm*(X) was not detected. In contrast, the resistance rate was lowest in the benzoyl peroxide monotherapy group (*n* = 117), at 9.4% (*n* = 11). Of these, 6.8% (*n* = 8) had 23S rRNA mutations and 2.6% (*n* = 3) had *erm*(50) but *erm*(X) was not detected. The frequencies of 23S rRNA mutations and *erm*(50) genes differed significantly between the clindamycin and benzoyl peroxide monotherapy groups (*p* < 0.05).

**TABLE 1:** History of topical antibbiotics use in acne patients and the rate of CLDM resistance and resistance factors in *C. acnes* isolated from patients.

Drugs	Number of users (*n*)	Resistance (*n*, %)	Resistant factor (*n*, %)		
			23S rRNA mutation	*erm* (X)	*erm* (50)
Benzoyl peroxide	117	11 (9.4)**	8 (6.8%)*	0	3 (2.6%)*
Clindamycin	30	14 (46.7)	6 (20.0%)	4 (13.3%)	4 (13.3%)
Clindamycin/Benzoyl peroxide	35	8 (22.9)*	2 (5.7%)	0	6 (17.1%)

*Note:* Based on a questionnaire survey of 182 acne patients from multiple clinical sites, topical antibiotic use was categorized into three groups: benzoyl peroxide monotherapy, clindamycin monotherapy, and clindamycin/benzoyl peroxide combination. The number of patients and corresponding *C. acnes* isolates in each group is shown. *p < 0.05, **p < 0.01 versus CLDM group.

### 3.2 Frequency of Emergence of Resistant Strains

**FIGURE 1:**
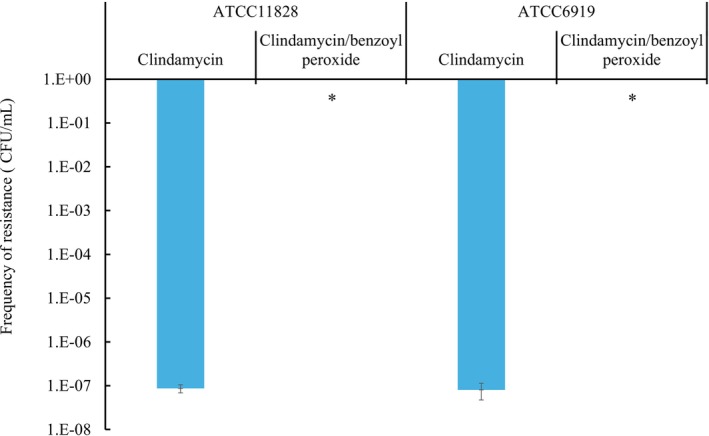
Frequency of occurrence of clindamycin‐resistant mutant in *Cutibacterium acnes*. Standard susceptible strains of *C. acnes* (ATCC 6919 and ATCC 11828) were inoculated onto agar plates containing clindamycin alone or clindamycin/benzoyl peroxide and cultured for 48 h. Colony‐forming units (CFU)/mL were counted to evaluate the frequency of resistance emergence. Data represent the mean ± standard deviation of three independent experiments. Statistical analysis was performed using Student's *t*‐test, with *p < 0.05 considered significant.

*C. acnes* reference strains were exposed to modified GAM agar plates containing either clindamycin alone or clindamycin/benzoyl peroxide (Figure 1). The frequency of clindamycin‐resistant mutants ranged from 4.9 × 10^−8^ to 1.5 × 10^−7^ CFU/mL for ATCC 6919 and from 6.0 × 10^−8^ to 1.2 × 10^−7^ CFU/mL for ATCC 11828 in the clindamycin alone group. All obtained resistant isolates harbored mutations in the 23S rRNA gene. In contrast, no resistant colonies were detected for ATCC 6919 or ATCC 11828 in the clindamycin/benzoyl peroxide group. These results indicated that the frequency of resistance emergence was significantly lower in the clindamycin/benzoyl peroxide group than in the clindamycin‐alone group (*p* < 0.05).

## 4 Discussion

We investigated the relationship between the use of topical agents (clindamycin, benzoyl peroxide, and clindamycin/benzoyl peroxide) and susceptibility of *C. acnes* isolates obtained from patients with acne vulgaris to elucidate the efficacy of clindamycin/benzoyl peroxide. Additionally, we evaluated the frequency of clindamycin‐resistant mutants in vitro after exposure to these agents to assess the potential risk of developing resistance during long‐term use.

We assessed the association between the history of drug use and clindamycin resistance using patient questionnaires. The clindamycin resistance rate was highest in the clindamycin monotherapy group. This was followed by that in the clindamycin/benzoyl peroxide and benzoyl peroxide monotherapy groups. There was a statistically significant difference between the groups (*p* < 0.05; Table 1). Therefore, clindamycin monotherapy may contribute to the development of clindamycin resistance. The clindamycin/benzoyl peroxide combination is considered beneficial for treating acne because of its synergistic antibacterial and anti‐inflammatory activity. However, according to the 2023 Guidelines for the Treatment of Acne Vulgaris and Rosacea, prolonged use of clindamycin is not recommended because of the potential risk of resistance development [4]. Furthermore, patients with a history of clindamycin treatment exhibit a higher prevalence of resistant isolates. Recent studies have reported an increasing prevalence of clindamycin‐resistant *C. acnes* strains in Japan [14]. Thus, we evaluated the frequency at which resistant mutants emerged in vitro after *C. acnes* exposed to clindamycin or clindamycin/benzoyl peroxide. Clindamycin‐resistant colonies were observed after exposure to clindamycin. However, no resistant colonies were detected after exposure to clindamycin/benzoyl peroxide. These findings strongly suggested that the addition of benzoyl peroxide suppressed the emergence of clindamycin‐resistant mutants.

Overall, the findings of this study demonstrate that monotherapy with clindamycin is strongly associated with the emergence of clindamycin‐resistant *C. acnes*, whereas the use of clindamycin/benzoyl peroxide may effectively suppress the development of resistance. Therefore, the concomitant use of benzoyl peroxide is strongly recommended in topical acne therapy to maintain antimicrobial efficacy against *C. acnes*. These findings support the current Japanese treatment guidelines, which emphasize combination therapy with topical agents [15]. However, further large‐scale clinical studies and comprehensive molecular epidemiological analyses are required to strengthen the existing evidence.

## Funding

This work was supported by JST SPRING Grant Number (JPMJSP2134) and Sun Pharmaceutical Industries Limited Inc.

## Conflicts of Interest

The author H.N. has financial conflicts of interest with Sun Pharmaceutical Industries Limited Inc., as the recipient of joint research funding. The other authors declare no conflicts of interest.

## Supporting information


**Table S1:**. Annual Detail of Topical Antibiotic use, CLDM Resistance Rates, and Resistance Factors in *C. acnes* from Acne Patients.

## Data Availability

The data that support the findings of this study are available on request from the corresponding author. The data are not publicly available due to privacy or ethical restrictions.
