# Supplementary material for: Combination Effects of Clindamycin and Benzoyl Peroxide Against Cutibacterium acnes
Source: J Dermatol. 2026 Feb 9;53(3):528–31. doi: 10.1111/1346-8138.70170 (PMC12967766; doi:10.1111/1346-8138.70170)
Supplement: Supplementary file 1 — Table S1:. Annual Detail of Topical Antibiotic use, CLDM Resistance Rates, and Resistance Factors in C. acnes from Acne Patients. [file JDE-53-528-s001.docx]

|  |  |  |  |  |  |  |
| --- | --- | --- | --- | --- | --- | --- |
| **Year** | **Drugs** | **Number of users (n)** | **Resistance (n, %)** | **Resistant factor (n, %)** | | |
|  |  |  |  | **23S rRNA mutation** | ***erm*(X)** | ***erm*(50)** |
| **2014** | **BPO** | **1** | **0 (0)** | **0** | **0** | **0** |
|  | **CLDM** | **0** | **0 (0)** | **0** | **0** | **0** |
|  | **CLDM/BPO** | **0** | **0 (0)** | **0** | **0** | **0** |
| **2015** | **BPO** | **0** | **0 (0)** | **0** | **0** | **0** |
|  | **CLDM** | **0** | **0 (0)** | **0** | **0** | **0** |
|  | **CLDM/BPO** | **0** | **0 (0)** | **0** | **0** | **0** |
| **2016** | **BPO** | **28** | **5 (17.9)** | **4 (14.2)** | **0** | **1 (3.6)** |
|  | **CLDM** | **10** | **4 (40.0)** | **4 (40.0)** | **0** | **0** |
|  | **CLDM/BPO** | **9** | **0 (0)** | **0** | **0** | **0** |
| **2017** | **BPO** | **36** | **5 (13.9)** | **4 (11.1)** | **0** | **1 (2.8)** |
|  | **CLDM** | **14** | **6 (42.9)** | **2 (14.3)** | **3 (21.4)** | **1 (7.1)** |
|  | **CLDM/BPO** | **18** | **7 (38.9)** | **2 (11.1)** | **0** | **5 (27.7)** |
| **2018** | **BPO** | **0** | **0 (0)** | **0** | **0** | **0** |
|  | **CLDM** | **0** | **0 (0)** | **0** | **0** | **0** |
|  | **CLDM/BPO** | **0** | **0 (0)** | **0** | **0** | **0** |
| **2019** | **BPO** | **2** | **0 (0)** | **0** | **0** | **0** |
|  | **CLDM** | **0** | **0 (0)** | **0** | **0** | **0** |
|  | **CLDM/BPO** | **0** | **0 (0)** | **0** | **0** | **0** |
| **2020** | **BPO** | **6** | **0 (0)** | **0** | **0** | **0** |
|  | **CLDM** | **0** | **0 (0)** | **0** | **0** | **0** |
|  | **CLDM/BPO** | **1** | **0 (0)** | **0** | **0** | **0** |
| **2021** | **BPO** | **0** | **0 (0)** | **0** | **0** | **0** |
|  | **CLDM** | **2** | **2 (100)** | **0** | **0** | **2 (100)** |
|  | **CLDM/BPO** | **0** | **0 (0)** | **0** | **0** | **0** |
| **2022** | **BPO** | **7** | **0 (0)** | **0** | **0** | **0** |
|  | **CLDM** | **1** | **0 (0)** | **0** | **0** | **0** |
|  | **CLDM/BPO** | **1** | **0 (0)** | **0** | **0** | **0** |
| **2023** | **BPO** | **37** | **1 (2.7)** | **0** | **0** | **1** |
|  | **CLDM** | **3** | **2 (66.7)** | **0** | **1 (33.3)** | **1 (33.3)** |
|  | **CLDM/BPO** | **6** | **1 (16.7)** | **0** | **0** | **1 (16.7)** |

CLDM, clindamycin; BPO, benzoyl peroxide.

**Supplement Table S1**. **Annual Detail of Topical Antibiotic use, CLDM Resistance Rates, and Resistance Factors in *C. acnes* from Acne Patients.**
